# P-1644. Real-World Effectiveness of Moderna’s mRNA-1273 COVID-19 Vaccine Across Multiple Seasons and Variant Periods in the United States

**DOI:** 10.1093/ofid/ofaf695.1820

**Published:** 2026-01-11

**Authors:** Amanda Wilson, Chris Clarke, Keya Joshi, Gigi Zheng, Nevena Vicic

**Affiliations:** Moderna, Inc., Cambridge, Massachusetts; Moderna, Inc., Cambridge, Massachusetts; Moderna, Inc, Cambridge, Massachusetts; Moderna, Inc., Cambridge, Massachusetts; Moderna, Inc, Cambridge, Massachusetts

## Abstract

**Background:**

The continued evolution of SARS-CoV-2 has led to annual reformulations of COVID-19 vaccines to maintain protection against emerging variants. Updates to Moderna’s mRNA-1273 vaccine (Spikevax) have included a bivalent (Wuhan-Hu-1 + Omicron BA.4/5) formulation in 2022, a monovalent XBB.1.5 formulation in 2023, and a monovalent KP.2 formulation in 2024. Evaluating real-world vaccine effectiveness (VE) of COVID-19 vaccines across these seasons, interpreted as incremental effectiveness against a background of immunity from prior vaccination and infection, is critical to understanding their public health impact and guiding ongoing vaccination strategies.

**Figure:**
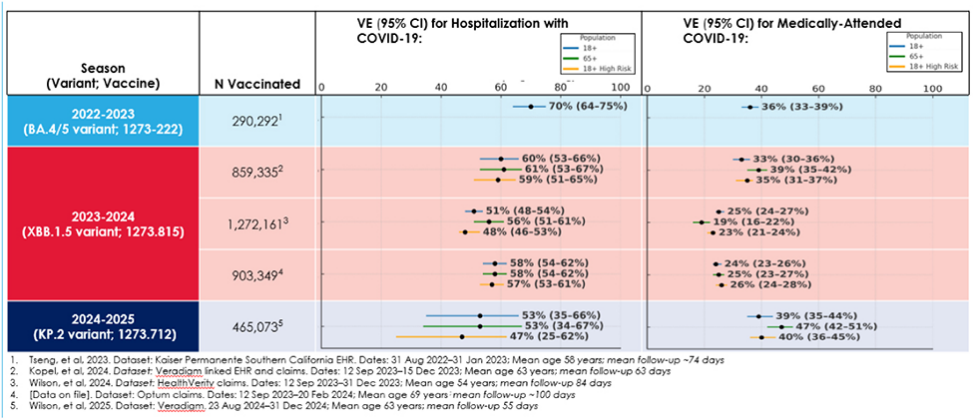
Vaccine effectiveness of Spikevax (mRNA-1273) against COVID-19-associated hospitalization and medically attended COVID-19, 2022–2025.

**Methods:**

We share findings from five retrospective matched cohort studies with similar methodology in U.S. electronic health record and administrative claims databases that focused on the VE of mRNA-1273 specifically. Each study compared adults vaccinated with mRNA-1273 to controls had not received a COVID-19 vaccine during the same respiratory virus season. Exact match, and multivariable adjustment or propensity score weighting were used to account for measured confounding. Potential confounders with absolute standardized difference > 0.1 were included in the adjusted models. VE was estimated as 1 minus the adjusted hazard ratio from Cox proportional hazards models, with outcomes including COVID-19-associated hospitalization and medically attended COVID-19.

**Results:**

Across seasons and formulations, VE in adults aged ≥18 years ranged from 51% to 70% against COVID-19-associated hospitalization and from 24% to 39% against medically attended COVID-19 (See Figure). VE estimates were similar for adults aged ≥18 years, aged ≥65 years, and aged ≥18 years at high risk of severe outcomes.

**Conclusion:**

The effectiveness of Moderna's updated mRNA-1273 vaccine formulations against severe COVID-19 outcomes remained stable across the most recent three respiratory virus seasons in the United States. These findings support the role of annual COVID-19 vaccination in reducing hospitalization and healthcare burden.

**Disclosures:**

Amanda Wilson, PhD, Moderna, Inc.: Employee|Moderna, Inc.: Stocks/Bonds (Public Company) Chris Clarke, PhD, Moderna, Inc.: Employee|Moderna, Inc.: Stocks/Bonds (Public Company) Keya Joshi, PhD, Moderna, Inc.: Employee|Moderna, Inc.: Stocks/Bonds (Public Company) Gigi Zheng, MD, PhD, ModernaTX: Employee|ModernaTX: Stocks/Bonds (Public Company) Nevena Vicic, MSc, Moderna, Inc.: Employee|Moderna, Inc.: Stocks/Bonds (Public Company)

